# Developing Digital Interventions: A Methodological Guide

**DOI:** 10.1155/2014/561320

**Published:** 2014-02-04

**Authors:** Katherine Bradbury, Sam Watts, Emily Arden-Close, Lucy Yardley, George Lewith

**Affiliations:** ^1^School of Psychology, University of Southampton, Southampton SO17 1BJ, UK; ^2^Primary Medical Care, University of Southampton, Southampton SO17 1BJ, UK

## Abstract

Digital interventions are becoming an increasingly popular method of delivering healthcare as they enable and promote patient self-management. This paper provides a methodological guide to the processes involved in developing effective digital interventions, detailing how to plan and develop such interventions to avoid common pitfalls. It demonstrates the need for mixed qualitative and quantitative methods in order to develop digital interventions which are effective, feasible, and acceptable to users and stakeholders.

## 1. Introduction

Healthcare resources are currently being put under pressure by ageing populations and increases in long-term health conditions [1]. There are growing calls for patients to self-manage their health and self-care is set to play a leading role in future healthcare [1, 2]. One potentially fruitful and increasingly popular method of promoting self-care and self-management is with digital interventions (DIs). DIs can offer self-care information, education, and behavioural support to patients remotely utilising technological mediums such as the Internet, mobile applications, and text messaging services. They can also allow healthcare practitioners to check a patient's progress. Evidence from a meta-analysis of 85 studies indicates that DIs can be effective for health management purposes, producing small but significant effects [3].

DIs can be extremely cost-effective [4], as once created they can be used for an infinite number of times by new users, unlike practitioner delivered interventions, which require staffing and room hire for each treated patient. DIs can complement practitioner delivered interventions by delivering routine aspects of healthcare, or providing self-care education and support for changing health behaviours, therefore allowing practitioners to focus on more complex tasks. DIs also offer benefits over paper based interventions, which require the production, printing and distribution of costly hard copy material. In addition to lowering costs, DIs can increase access for users, both by providing 24-hour access to healthcare interventions and increasing access to healthcare for those who might find it harder to attend appointments (e.g., because of their location or health problem) [4].

DIs have the potential to reach huge numbers of people. Recent research suggests that most households in the Western World now have access to the Internet [5]; over 71% of households have accessed the Internet in the United States [6] and 86% in the United Kingdom [7]. Likewise, over 90% of the population in these countries currently have access to and use mobile phones [8, 9]. Internet and mobile phone access in the developing world is also growing rapidly, for instance, 15.9% of African households now have access to the Internet [10] and by 2012 54% owned mobile phones (although since many people share mobiles, access to one is likely to be higher) [11].

In the past DIs have been costly and difficult to create, requiring expertise in programming. This changed with the introduction of LifeGuide (https://www.lifeguideonline.org/), a University of Southampton initiative, which allows people with no programming knowledge to easily create DIs using free software [12]. The pages of a DI can include text, pictures, or videos and a series of pages can be shown one after another to produce a session. For example, a user might complete a session on healthy eating which includes several pages of information about particular food groups and then a guide to setting goals to change eating habits. Questionnaires can also be included, which can help collect data for research and also tailor intervention content to users (for instance by gender, user preferences, or health risks), thereby showing users information which is most relevant to them. LifeGuide is also able to send remote prompts to users in the form of personalised emails and text messages and can provide a facility for healthcare professionals to monitor patient progress. Furthermore, this software collects both research data (from questionnaire responses) and usage data, enabling practitioners and researchers to keep track of a patient's progress through a DI.

Many DIs are developed to support mainstream healthcare practitioners, such as GPs or nurses (e.g., [13–15]). However, there are many instances where a DI might be developed to support or enhance face to face care in CAM therapies. For instance, a DI could enhance osteopathy for back pain by teaching patients exercises to strengthen their backs, thus freeing practitioners to focus on providing hands on techniques during treatment sessions. Nutritional advice and support (for instance, with weekly goal setting and reviewing) could support a number of CAM therapies such as acupuncture or Ayurveda, which sometimes offer nutritional advice alongside other techniques [16, 17]. Such DIs could help improve patients' adherence to lifestyle advice by teaching them skills to help make behaviour changes habitual. In other instances it may be possible for some CAM modalities to be delivered primarily through a DI, eliminating the costs of face to face care. Modalities such as autogenic training, meditation, progressive muscle relaxation, guided imagery, and mindfulness based stress reduction are known to be effective in reducing stress, depression, anxiety, fatigue, and pain [18–21] and might be usefully incorporated into DIs. Indeed, some already have real success; for instance, face to face and online mindfulness interventions appear to be equally effective for reducing stress [22]. It is important to note that there may be particular CAM therapies which cannot be delivered partially or fully through a DI, such as the Alexander Technique, which requires the touch of a practitioner to guide patients through improvements in their posture.

Much of the literature concerning developing DIs is complex, technical, and not aimed at beginners, who require an overview of the methods involved. The methodological processes involved in creating mainstream and CAM DIs are the same; however, we are not aware of any literature aimed at a CAM audience. Our aim is to equip a CAM audience with the knowledge needed to embark on creating DIs. The current article will provide a guide to the methodological processes involved, outlining best practice and how to avoid potential pitfalls; it will cover both initial intervention planning and further intervention development with user testing.  Table 1 shows an overview of this process, including critical issues to consider at each stage.

## 2. Intervention Planning 

There is widespread consensus that systematic intervention planning incorporating existing evidence, theory, and the views of potential users (and those involved with their care) is key to creating interventions which will be successful and widely adopted into practice [1–7].

Several decisions must be made prior to starting work on a DI design. Firstly, the key behaviours that users need to perform in order to improve their health problem must be identified [13, 23–28]. For instance, users may need to make lifestyle changes, such as increasing their physical activity, or perhaps self-monitor their behaviour or a particular aspect of their health such as their weight or mood.

Secondly, it is important to consider the modality through which a DI will be delivered; common modalities include computers, smartphones, or simply text messages. The nature of the intervention that can be produced using each of these modalities will be constrained by the capabilities of each technology. For instance, computer based interventions can include multiple sessions that users complete over time, therefore incorporating multiple and complex behaviour change techniques. Larger screens also mean that more detailed information can be included. In contrast Smartphone's provide a smaller screen, so less information can be displayed. However, Smartphone's could provide more accessible interventions than computers, since people tend to carry them with them most of the time and look at them frequently [29–32]. Smartphone technology also includes sensors, for instance, GPS, to recognise location and voice sensors which might be used to identify mood changes [29, 33–35]; research into how best to harness these sensors for intervention design is ongoing [29, 35]. It is possible that in the near future such sensors could enable intervention at critical time points, such as when users are lower in mood and might lack motivation to carry out positive health behaviours. Text messages are limited to providing the briefest of information, but are likely accessible to the widest range of individuals, including those in lower SES groups [36]. Text message interventions can include brief advice, support, or reminders to perform health behaviours. One study showed that smokers receiving smoking cessation advice, support, and distraction via text were twice as likely to quit compared to those in a control group [37].

Once key user behaviours and modality of delivery have been determined, likely influences on key behaviours can be identified [23, 28]. A combination of deductive and inductive approaches is useful at this point.

### 2.1. Deductive Approaches

Deductive approaches are useful to ascertain what is already known about changing a behaviour; these approaches include reviews of the existing literature and theory that might usefully inform intervention design.

Reviews of the existing quantitative literature (in particular RCTs) are useful to elicit what behaviour change techniques are likely to be most effective and cost effective. Such studies have high internal validity and are therefore capable of isolating cause and effect [38]. However, such studies cannot answer questions about how acceptable an intervention is to a user or why users fail to adhere to an intervention. Inductive qualitative research is instead able to answer such questions as it enables in depth exploration of users' viewpoints. Reviews of the qualitative literature focussing on participants' experiences of using particular behaviour change techniques or entire interventions are therefore equally valuable, providing insights into what intervention components are likely to be acceptable to users and feasible in practice. The downside of qualitative studies is that they lack the precision and control needed for high internal validity, meaning that they cannot draw conclusions about cause and effect or statistical relationships. The combination of both quantitative and qualitative evidence therefore means that the strengths of both methods can be utilised and the limitations of each are to some extent overcome by the inclusion of the other.

A number of intervention development guidelines recommend that theory should also inform intervention design, as it can improve specification of potentially active intervention components [23, 25]. Indeed, systematic review evidence shows that more extensive use of theory in DIs is associated with larger effect sizes [3]. Theory can inform DI developers of the likely influences on key behaviours. For example the Behaviour Change Wheel provides a system for identifying what individual level or environmental factors need to change in order for a user to perform a given behaviour [26]. The model links these key influences on behaviour to intervention functions (e.g., education, providing incentives, or modelling), meaning that once a key influence that is lacking is highlighted, the behaviour change functions necessary to increase that key influence on behaviour can be easily identified and implemented in order to change behaviour. Taxonomies of behaviour change techniques are also useful at this point, as they identify a wide range of techniques which can be usefully employed to enhance or minimise key influences on behaviour [39, 40].

### 2.2. Inductive Approaches

Equally valuable to DI planning is inductive qualitative work, which can provide important information about which intervention features might be acceptable (or unacceptable) to users and therefore which of those highlighted by deductive approaches might be most usefully integrated [13]. Inductive research is particularly valuable at the intervention planning stage if only limited qualitative literature assessing the acceptability of intervention components within the target population is available. Sam Watts and George Lewith have been developing a DI for men with prostate cancer assigned to active surveillance, a process which is known to be stressful [41]. At first deductive research suggested that a mindfulness meditation intervention would be an effective way of reducing stress. However, in early qualitative interviews it was identified that these patients held negative perceptions of mindfulness meditation and were not prepared to engage in such an intervention. The idea of a “stress reduction” intervention which incorporated the breathing exercises and relaxation techniques from mindfulness programmes was, however, viewed as acceptable. Qualitative work therefore highlighted how the intervention could be repackaged to appeal to men with prostate cancer.

The views of stakeholders, such as healthcare staff who might be supporting a DI or affected by its implementation, should also be considered to ensure that a DI is developed which is acceptable and feasible to adopt in practice [19, 27]. For example, focus groups with healthcare practitioners revealed that they have little time or training to deliver weight management services but that they would welcome a DI which supported patients in losing weight [42]. As staff had little training in weight management techniques, a DI was designed to provide the weight loss advice while practitioners were involved to support and encourage patients in order to help maintain patient motivation. DIs developed without consideration of the opinions of stakeholders and users are less likely to be successfully implemented [27].

A limitation of using an inductive approach in intervention development is that the small samples employed might not be representative of the entire target population and some viewpoints may therefore be missed. This problem can be reduced by seeking a maximum variation sample, which includes a range of people from the target population (e.g., from different backgrounds, genders, race, employment status, or stage of their particular health problem) so that a wider variety of viewpoints are likely to be captured [43]. Importantly, inductive qualitative research is able to capture novel responses which have not been anticipated by the researcher. In contrast, deductive research (e.g., questionnaire designs) requires the researcher to choose the possible responses that users will give in advance, meaning that vital information might not be captured [44], which could lead to problems with the acceptability of a DI being overlooked.

### 2.3. Integrating Deductive and Inductive Approaches: Creating an Intervention Plan

Once completed, the findings of the deductive and inductive research need to be collated to create a plan of what the intervention should contain in order to maximise effectiveness, acceptability, and feasibility. Decisions can then be made about when each intervention component should be introduced to the user; for instance it might be important to introduce goal setting early on, whereas environmental restructuring might need to be addressed later.

It is also useful at this stage to plan the practical elements of the DI, which can influence uptake. For example, potential users may fail to register because of concerns about the privacy or security of their data. A security page at the beginning of an intervention can allay concerns and avoid problems with uptake. Such a page should include information about who has access to data (e.g., the research team), that data is password protected, anonymised, and protected by firewalls [20]. The process of initially signing up for a DI and then later logging back on can also confuse users and mean they end up registering multiple times, therefore starting an intervention again rather than logging back into where they were last time. This can be resolved by making log in buttons larger than sign up buttons, so that users will be more likely to log in and avoid signing up multiple times. The LifeGuide beginner's guide [20] is a useful resource for planning practical aspects of a DI.

### 2.4. Prioritisation: Deciding What Makes the Final Cut

Whilst researching and planning the aspects of an intervention likely to be the most feasible, acceptable, and effective for users, it is also vital to consider what will be feasible for the development team. Time and staffing resources are likely to be limited for most DI developers, prioritisation of the components which are the most essential to a particular intervention is therefore necessary [45, 46]. Prioritisation should be adjusted throughout intervention planning and development phases, as unexpected issues arise; this is therefore best considered an iterative process. MoSCoW, a prioritisation model used by digital service developers [46, 47], is a simple tool that can be usefully employed to prioritise the content of DIs. Each of the capital letters represents a priority:

- *M: Must have* this for the intervention to be effective, acceptable and feasible,
- *S: Should have* (if possible) for the intervention to be a success, but may be able to be delivered in a different way, or is in some way less critical than a Must have,
- *C: Could have* this as it would be useful, but only if time and resources are available,
- *W: Would like* for classifying a feature which would be nice, but is not essential now and can be put on hold.

In our experience this tool ensures efficient development of successful interventions and aids effective communication between team members, who might otherwise perceive different priorities.

## 3. Intervention Development 

Once intervention plans have been put into place and a DI created, usability testing can be employed to further develop and improve the DI. Usability testing originated in the field of human-computer interaction but is now considered essential in the development of e-health interventions [48]. It is used to assess the target group's views regarding the content and format of the intervention during development and evaluate user experiences following completion of the intervention [49]. It aims to identify and eliminate barriers to easy and efficient use by members of the target population and to establish user acceptability and satisfaction with the intervention [49]. Usability testing is an iterative process: it leads to changes being made to the DI, which are then followed by further user testing. This process continues until no further suggestions for changes are being made (e.g., [50]). There are two main qualitative methods for carrying it out: think-aloud interviews, which involve asking users to vocalise their reactions and thinking processes while they use the DI and retrospective semistructured interviews [51]. Each method is discussed in more detail below.

### 3.1. Think-Aloud Interviews

During think-aloud interviews, participants, with the researcher present, are observed using the DI and asked to comment on their reactions to every aspect of the intervention. In the case of computer based interventions with several sessions, a participant might look at one or two sessions during each think-aloud interview, meaning multiple interviews will be required to test the whole intervention. Think aloud interviews enable researchers to identify problems people might experience when carrying out the intervention and modify the DI accordingly. They are extremely effective in highlighting navigational difficulties [52] and can reveal useful information relating to the content of DI which needs to be modified [50]. For example, think-aloud studies investigating a web-based weight loss intervention revealed that participants did not always implement goal setting and planning as intended. They often restated goals or made imprecise or irrelevant plans which would not achieve weight loss [13]. Following these interviews, the researchers therefore modified the web site by supplying concrete examples of suitable goals, with preset goal choices [13]. These were sufficiently specific and challenging to achieve weight loss if followed and gave more detailed illustrations of how to make plans.

### 3.2. Retrospective Interviews

Retrospective semistructured interviews are valuable to evaluate user experiences after completion of part of, or the entire, DI. Here, participants try out the intervention alone as an end user and are interviewed about their experiences after completing some or all of the intervention. Retrospective interviews complement think-aloud methods by providing information about how people use the DI in the absence of a researcher, who might otherwise influence participants experiences of carrying out the intervention [53]. For instance, participants might look at different information or use the DI in a different way when not being observed. Furthermore, using the intervention alone allows participants to try out new behaviours recommended by the DI, such as making dietary changes, which can then provide useful information about how well the DI supports users in making such changes. Drawbacks of this method include attrition over the course of the intervention, and the logistics of carrying out interviews before too much time has elapsed following completion of the intervention [54]. This is particularly important with regard to health conditions where the condition or treatment might impair long-term memory. Asking participants to keep a diary of their experiences of using the DI could help to overcome problems of forgetting important information about using a DI. We have found it useful to ask participants to note any aspects that they found particularly useful or not useful, easy to use or problematic and aspects which they particularly liked or disliked. In addition, retrospective data can be triangulated with data collected by the DI itself about how the participant has used the intervention (for instance what pages they have viewed).

Think aloud and retrospective approaches should be seen as complementary, not competing. They offer additional data which can be usefully triangulated for a fuller picture of how people use a DI and any improvements which might be usefully made [55]. These two approaches might also be best employed at different time points in the development phase; whilst think-aloud approaches are useful early on to iron out major content or navigational issues, retrospective studies can be helpful later to identify any further problems occurring when people use the intervention alone.

Once an initial DI appears acceptable to users, it can then enter into a feasibility study, a small scale randomised clinical trial (RCT) designed to test the processes of carrying out a fuller trial in a smaller (and therefore less costly) group of patients. Such designs are extremely useful for highlighting any problems with the feasibility of using a DI in the context that it will eventually be implemented into [56]. They can also estimate sample sizes needed to test an intervention based on effect sizes [56], although they cannot test whether an intervention is effective as they are inevitably underpowered to do so. Data from feasibility studies can then be used to make final refinements to the DI before its effectiveness is tested in a fully powered RCT. The Medical Research Council (MRC) provides extremely useful guidance on testing complex interventions (such as DIs) in RCTs [23, 57]. Retrospective interviews carried out at the end of both feasibility and fully powered RCTs can further enhance interpretation, highlighting explanations for quantitative results or further ways in which a DI might be improved.

Once a DI has been shown to be effective, feasible, and acceptable to users, implementation studies can be useful to determine how best to implement the DI into routine practice, to identify any unintended adverse effects, and to test whether the DI is equally as effective in this environment [23, 57]. Carrying out qualitative research throughout the planning and development stages, considering and responding to the views of both end users and stakeholders (such as healthcare professionals), as outlined in this article, should mean that problems with implementation can be largely avoided [27].

## 4. Conclusion

The methodological processes involved in developing DIs have been outlined; we have demonstrated how a mixture of qualitative and quantitative designs can be used to ensure the effectiveness, feasibility, and the acceptability of DIs to both end users and stakeholders. The technological capabilities of DIs are increasing at a rapid pace and it is likely that they will become an increasingly important component of healthcare. It is hoped that this guide will help to promote the rigorous development of DIs and encourage the CAM community to take advantage of the technology available, in order to enhance patients' empowerment and effective self-management of their problems.

## Figures and Tables

**Table 1:** Key steps and common critical issues in developing DIs.

Key steps	Common critical issues
Intervention planning	
What are the key behaviours to be targeted?	

What modality is most appropriate for delivery (e.g., computers, smartphones, or text messages)?	

Determine likely influences on key behaviours using (i) deductive reviews of the literature and implementation of theory, (ii) inductive research with users and stakeholders.	Ensure both quantitative and qualitative literatures are reviewed to fully understand likely effectiveness and acceptability of DI components.

Create an intervention plan. (i) What should the intervention contain to maximise effectiveness, acceptability, and feasibility? (ii) When should each intervention component be introduced to the user? (iii) Plan practical aspects of the DI (such as security and logging on procedures).	Include a security page to allay user concerns. Make the logging on button larger than the register button to avoid problems with users registering multiple times.

Prioritise intervention components to ensure feasibility for the development team.	

Intervention development and usability testing	
(i) Think-aloud interviews to assess user perceptions and interactions with the intervention. (ii) Modify the DI as needed.	Be sure to observe how users navigate the intervention during think-aloud interviews as this provides additional information about potential problems.

Retrospective interviews with users who have tried the intervention alone.	Try to conduct interviews within a week of the participant using the DI to maximise recall. Users can keep a diary of their experiences of using the DI to improve recall during interviews.

(i) Triangulate user data (e.g., aspects of DI viewed) with retrospective interview data to gain a fuller picture of how the DI is used when participants are alone. (ii) Modify the DI as needed.	

Intervention testing	
Feasibility RCT to test the study processes for a full trial.	Retrospective interviews can highlight ways to improve study processes or the DI for the main trial.

Fully powered RCT to test the effectiveness and cost-effectiveness of the DI.	Retrospective interviews can enhance interpretation of quantitative results.
